# *Babesia* sp. EU1 Infection in a Forest Reindeer, the Netherlands

**DOI:** 10.3201/eid1705.101834

**Published:** 2011-05

**Authors:** Marja Kik, Ard M. Nijhof, Jesper A. Balk, Frans Jongejan

**Affiliations:** Author affiliations: Utrecht University, Utrecht, the Netherlands (M. Kik, A.M. Nijhof, J.A. Balk, F. Jongejan);; University of Pretoria, Onderstepoort, South Africa (F. Jongejan)

**Keywords:** Babesia sp. EU1, parasites, babesiosis, Rangifer tarandus fennicus, forest reindeer, zoonosis, the Netherlands, letter

**To the Editor**: Fatal piroplasmosis in domestic reindeer (*Rangifer* spp.) was first reported by Kertzelli in 1909; he named the piroplasm *Piroplasma tarandi rhangferis*. Similar piroplasms also were observed in blood smears of reindeer that had a condition known as spleen disease, which occurred in the second part of summer in the Arctic tundra and was characterized by clinical signs such as splenomegaly, icterus, pale mucous membranes, and death (*1*). Hemoglobinuria, a characteristic sign of babesiosis, is not mentioned in these early 20th century reports. However, these signs were observed in a *Babesia divergens*–infected reindeer herd in Scotland (*2*).

The only other reported cases of severe babesiosis in reindeer and caribou (*Rangifer tarandus caribou*) were caused by *B. odocoilei*, a predominantly nonpathogenic parasite of white-tailed deer (*Odocoileus virginianus*) that can cause fatal infection in reindeer (*3**,**4*). *Babesia* sp. EU1 is a recently recognized zoonotic *Babesia* species that has been associated with human babesiosis in Europe and is phylogenetically related to the *B. odocoilei* parasite (*5*). We report on a juvenile reindeer with babesiosis caused by *Babesia* sp. EU1.

A 5-week-old, captive-bred, female forest reindeer from an otherwise healthy herd of 9 animals in a zoo in the Netherlands was euthanized after showing clinical signs of lethargy, jaundice, and hemorrhagic diarrhea for >8 hours that did not improve after treatment with butylscopolamine (Buscopan; Boehringer Ingelheim, Alkmaar, the Netherlands) and enrofloxacin (Baytril; Bayer, Leverkusen, Germany). At necropsy, jaundice was evident in the sclera, aorta, and leptomeninges. On the basis of the degree of fat storage and muscle development, the body condition was fair. The lungs were hyperemic and edematous, and the trachea contained foam. The liver was enlarged and pale; the spleen was enlarged. The kidneys were dark brown. Hemoglobinuria was noted in the urinary bladder.

Tissue samples from various organs were in fixed in 4% phosphate-buffered formalin, embedded in paraffin, cut into 4-µm sections, and stained with hematoxylin and eosin. No microscopic lesions were found in the skin, thymus, thyroid gland, tonsils, salivary glands, tongue, gastrointestinal tract, or heart. Numerous hemosiderin-laden macrophages were found in the spleen and liver sinusoids. Pigmentary nephrosis with moderate tubular degeneration and focal interstitial petechial hemorrhages were seen in the kidneys. Erythrophagocytosis was evident in the mesenteric lymph nodes, liver, and spleen.

Cytologic analysis was performed on samples from the brain, liver, spleen, lungs, and large intestinal contents, which were stained with Hemacolor (Merck, Darmstadt, Germany). Large (2–3 μm), intraerythrocytic protozoal inclusions consistent with *Babesia* spp. (Figure) were identified in the liver, spleen, lung, and brain.

**Figure:**
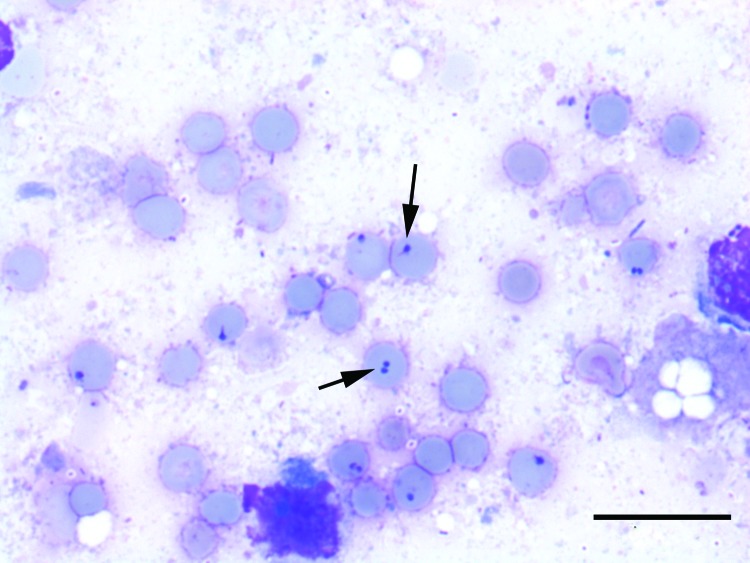
Lung of a forest reindeer infected with *Babesia* sp. EU1. Arrows indicate erythrocytes with protozoal inclusions. Scale bar = 20 µm.

DNA was extracted from 200 µL blood and ≈25 mg of the tissues collected during the necropsy: bone marrow, brain, heart, kidney, liver, lung, lymph node, small intestinal wall, and spleen. All extractions were performed by using the Nucleospin Tissue Kit (Macherey-Nagel, Düren, Germany) according to the manufacturer’s protocol. The detection of PCR products by reverse line blot hybridization was performed as described (6). All blood and tissue samples from each organ tested were positive only for *Babesia* sp. EU1.

To confirm these results, primers 18SAN and 18SBN were used to amplify a 1,705-bp fragment of the 18S rRNA gene (*7*), the fragment was subsequently purified, cloned into the pGEM-T Easy Vector (Promega, Leiden, the Netherlands), and sequenced (Baseclear, Leiden, the Netherlands). The resulting sequence (GenBank accession no. GQ888709) was 100% identical to that of *Babesia* sp. EU1 isolated from human babesiosis patients from Italy and Austria (GenBank accession no. AY046575). In an attempt to identify subclinical carriers of this piroplasm, blood samples from the reindeer’s mother and another nonrelated calf from the herd were collected and tested by reverse line blot, but test results for both animals were negative.

These findings make transplacental transmission as a route of infection less likely and favor the bite of an infected tick as the cause of disease. *Babesia* sp. EU1 is transmitted by *Ixodes ricinus* ticks (*8*), which are widespread in cool humid areas of Europe. Of *I. ricinus* ticks from the Netherlands, ≈1% are infected with *Babesia* sp. EU1 (*6*). The only confirmed reservoir host of *Babesia* sp. EU1 is roe deer (*Capreolus capreolus*) (*9*). The infected forest reindeer resided in a zoo in an area without direct contact with roe deer, although roe deer are abundant in the forests surrounding the zoo.

Because *Babesia* sp. EU1 can be transmitted both transovarially and transstadially (*10*), the infection source may have been the offspring of a tick infected in a previous generation or an immature tick that fed on infected roe deer outside the zoo or an as yet unidentified reservoir host and was carried into the reindeer’s compound by hosts, such as birds or small rodents. *Babesia* sp. EU1 is the third *Babesia* spp. to be recognized as the cause of fatal babesiosis in reindeer, together with *B. divergens* and *B. odocoilei*.
